# MICU1 protects against myocardial ischemia/reperfusion injury and its control by the importer receptor Tom70

**DOI:** 10.1038/cddis.2017.280

**Published:** 2017-07-13

**Authors:** Qiang Xue, Haifeng Pei, Qinshe Liu, Mingjun Zhao, Jing Sun, Erhe Gao, Xinliang Ma, Ling Tao

**Affiliations:** 1 Department of Cardiology, Xijing Hospital, Fourth Military Medical University, Xi’an 710032, China; 2 Department of Cardiology, Chengdu Military General Hospital, Chengdu 610083, China; 3 Shaanxi University of Chinese Medicine, Xi’an 712046, China; 4 Center of Translational Medicine, Temple University School of Medicine, Philadelphia, PA 19140, USA; 5 Department of Emergency Medicine, Thomas Jefferson University, Philadelphia, PA 19107, USA

## Abstract

Mitochondrial Ca^2+^ overload is a main contributor to mitochondrial damage hence cardiomyocyte death in myocardial ischemia/reperfusion (MI/R) injury. MICU1 has been recently identified as an important regulator of mitochondrial Ca^2+^ homeostasis. Here we try to identify the role of MICU1 in MI/R, and to investigate whether the mitochondrial importer receptor Tom70 possesses critical roles in the mitochondrial translocation of MICU1 and MI/R. Specific small interfering RNA (20 *μ*g) against MICU1 and Tom70, and lentivirus vectors carrying the *Tom70a* sequences (3.3 × 10^7^ TU) were delivered through intramyocardial injection. Seventy-two hours after injection, mice were subjected to 30 min of MI followed by 3 h (for cell apoptosis and mitochondrial damage assessment) or 24 h (for cardiac function and infarct size determination) of reperfusion. MI/R had no significant effect on total MICU1 expression, but caused significant reduction of MICU1 in mitochondria. Knockdown of MICU1 significantly aggravated MI/R injury, as evidenced by enlarged infarct size, depressed cardiac function and increased myocardial apoptosis. Moreover, MICU1 deficiency resulted in markedly aggravated mitochondrial Ca^2+^ overload, consequently destructed mitochondrial morphology and suppressed mitochondrial function (evidenced by decreased ATP production). Interestingly, mitochondrial Tom70 was also decreased in MI/R. Genetic loss-function study revealed that mitochondrial MICU1 expression was depressed by Tom70 ablation. Furthermore, Tom70 deficiency significantly aggravated MI/R injury and worsened mitochondrial Ca^2+^ overload. However, supplementation of Tom70 significantly attenuated MI/R injury, preserved mitochondrial morphology and function, and inhibited mitochondrial Ca^2+^ overload, all of which were abolished by MICU1 suppression. Mitochondrial Tom70/MICU1 pathway protects against MI/R injury, in which mitochondrial localization of MICU1 is governed by Tom70, and MICU1 serves as an indispensable factor in Tom70’s cardioprotection.

Reperfusion strategies with the use of thrombolytic agents and primary percutaneous coronary intervention is undoubtedly beneficial in myocardial infarction (MI), however, they also cause irreversible detrimental effects termed myocardial reperfusion injury.^1, 2^ Identifying novel therapeutic interventions reducing reperfusion injury may increase survival rate and ultimately reduce death rate caused by MI.

The structure and biochemical functions of mitochondria, the primary source of ATP supply in the contracting cardiac myocytes and the ‘headquarter’ of apoptotic cell death, are the major targets of ischemia/reperfusion (I/R) injury.^3, 4, 5^ Mitochondrial Ca^2+^ homeostasis has an important role in the maintenance of a variety of cellular functions.^6^ Ca^2+^ is central to the cardiac excitation–contraction coupling and the signaling networks that regulate pathological myocardial growth and remodeling.^7^ Accumulating evidence show that mitochondrial Ca^2+^ overload is associated with mitochondrial dysfunction, contractile dysfunction and cell death.^8, 9^ Complete understanding of the molecular mechanisms leading to the elevation of mitochondrial Ca^2+^ content in post-MI cardiomyocytes thus may hold great promise in attenuating myocardial ischemia/reperfusion (MI/R) injury.

Recent experimental evidence indicates that Ca^2+^ handling at mitochondrial level is more tightly controlled by the balance between molecules that stimulate mitochondrial Ca^2+^ uptake and molecules that inhibit Ca^2+^ uptake.^10^ Specifically, the mitochondrial Ca^2+^ uniporter (MCU) is the major molecule stimulating mitochondrial Ca^2+^ uptake.^11^ With an *in vivo* MI/R model, Luongo *et al.*^12^ revealed that MCU deficiency conferred resistance to IR injury by preventing mitochondrial Ca^2+^ overload. Moreover, mitochondrial Ca^2+^ uptake 1 (MICU1) was recently identified as a molecule localized to the inner mitochondrial membrane to regulate mitochondrial Ca^2+^ uptake.^13^ The MICU1 serves as an inhibitory effecter for mitochondrial Ca^2+^ influx with localization to the inner mitochondrial membrane.^14^ These two molecules biochemically interact with each other, and they are co-expressed across tissues and species.^15^ Mallilankaraman *et al.*^16^ found that MICU1 was an essential inhibitory gatekeeper for MCU-mediated mitochondrial Ca^2+^ uptake that regulated cell survival. MICU1 is required to preserve normal mitochondrial Ca^2+^ level under basal conditions. In its absence, mitochondria become constitutively loaded with Ca^2+^ through MCU, triggering excessive reactive oxygen species generation and increasing sensitivity to apoptotic stress.^16^ However, whether MI/R may alter MICU1 expression/function, thus contributing to post-MI mitochondrial Ca^2+^ overload and subsequent cell death, have not been previously investigated.

Although mitochondria possess their own genome that encodes for 13 essential subunits of the oxidative phosphorylation system, the majority of ~1500 mitochondrial proteins are synthesized in the cytosol and then transported into corresponding compartments in mitochondria. Breakthroughs on mitochondrial protein targets demonstrate that translocases of outer membrane (Tom) complex is responsible for initial recognition of mitochondrial pre-proteins from the cytosol.^17^ As an important receptor of the Tom machinery, Tom70 preferentially binds the internal targeting signals of polytopic membrane proteins.^18^ Recent studies demonstrated that Tom70 was essential for PTEN-induced kinase 1 (PINK1) import into mitochondria,^19^ which decreased the cardiac vulnerability to I/R injury.^19, 20^ However, how MICU1 is translocated from cytosol to the inner mitochondrial membrane of cardiomyocyte and whether Tom70 is involved in this process remain unknown. Moreover, whether the expression/function of Tom70 is inhibited in ischemic/reperfused cardiomyocytes, thus impairing MICU1 localization, has not been previously investigated.

Therefore, the aims of present study are (1) to determine whether MI/R may alter mitochondrial localization of MICU1; if so, (2) whether it may contribute to mitochondrial dysfunction and MI/R injury; and (3) to identify the molecular mechanisms, particularly Tom70, controlling subcellular localization of MICU1.

## Results

### Mitochondrial MICU1 expression is downregulated following MI/R, and MICU1 deficiency aggravated MI/R injury

**Figure 1:**
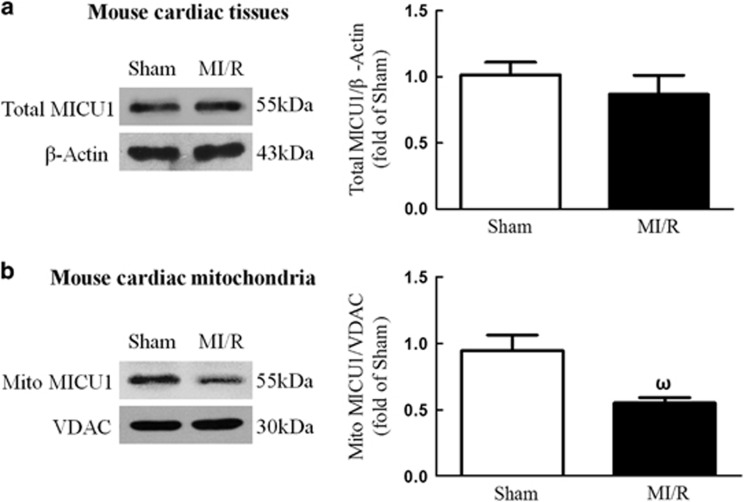
MICU1 responded to MI/R injury. (**a**) Representative western blot images showing total MICU1 expression using *β*-actin as loading control in the left panel; intensities of total MICU1 relative to *β*-actin in the right panel. (**b**) Representative western blot images showing mitochondrial MICU1 expression using VDAC as loading control in the left panel; intensities of mitochondrial MICU1 relative to VDAC in the right panel. Mito, mitochondria; VADC, voltage-dependent anion channel. Presented values are means±S.E.M. *N*=6–8/group. ^ω^*P*<0.05 *versus* sham

**Figure 2:**
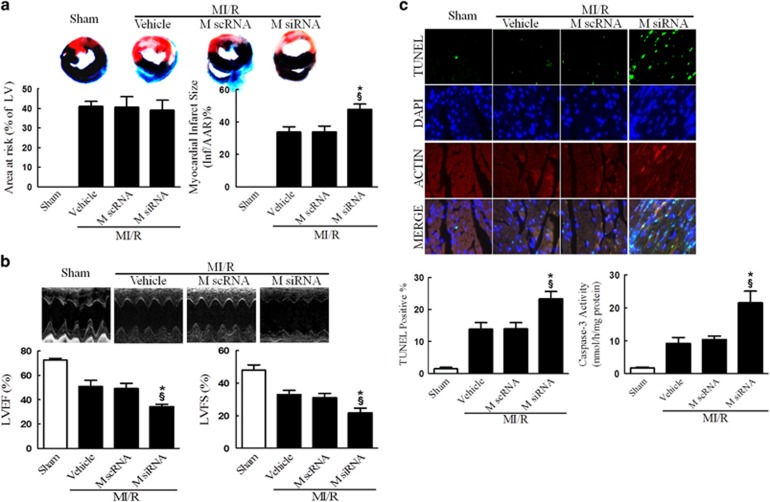
MICU1 deficiency worsened MI/R injury. (**a**) Myocardial infarct size was assessed by Evans blue/TTC double staining. The upper panel showed heart sections obtained from mice at 24 h after MI/R injury. Evans blue-stained areas (black) indicated non-ischemic/reperfused area; TTC-stained areas (red staining) indicated ischemic but viable tissue; Evans blue/TTC-staining-negative areas indicated infarct myocardium. The lower panels showed summary of area at risk (AAR) per left ventricle (LV) and infarct area (Inf) per AAR. (**b**) Cardiac function was assessed by echocardiography in mice 24 h after MI/R injury. Representative M-mode images were shown in the upper panel. Left ventricle ejection fraction (LVEF) and fractional shortening (LVFS) were showed in the lower panels. (**c**) Myocardial apoptosis was determined by TUNEL staining in the upper panel. TUNEL staining (green) indicates apoptotic nuclei; DAPI counterstaining (blue) indicates total nuclei. TUNEL-positive nuclei were expressed as a percentage of the total number of nuclei, automatically counted and calculated by Image-Pro Plus software in the left of lower panel. Myocardial apoptosis was determined by caspase-3 activity assay in the right of lower panel. M scRNA, scrambled siRNA used as control; M siRNA, MICU1-specific siRNA. Presented values are means±S.E.M. *N*=6–8/group. *P<0.05 *versus* vehicle of MI/R; ^§^*P*<0.05 *versus* M scRNA of MI/R

To determine the role of MICU1, a newly identified regulator of mitochondrial Ca^2+^ homeostasis, in MI/R, we first determined the total and mitochondrial MICU1 protein expression level following MI/R. Interestingly, I/R induced significant reduction in mitochondrial MICU1 expression, but not in total MICU1 expression, compared with sham-MI/R counterpart (Figure 1). To determine the functional significance of MICU1 inhibition following MI/R, mice models with cardiac MICU1 knockdown were successfully established by intramyocardial injection of MICU1-targeted small interfering RNA (siRNA; Supplementary Figures 1A and B). Although MICU1 downregulation slightly increased mitochondrial Ca^2+^ content in cardiomyocytes, however it did not significantly disturb mitochondrial respiration, nor did it induce obvious myocardial injury (Supplementary Figures 1C–F). Moreover, we did not find any changes in basal physiological parameters in MICU1 knockdown mice (Table 1). Using ELISA kit, 2, 3, 5-triphenyltetrazolium chloride (TTC) staining and ultrasonography, we found that MICU1 deficiency markedly enhanced MI/R-induced cardiac troponin-I (cTnI) leak, MI and contractile dysfunction (Figures 2a and b and Supplementary Figure 2). The evaluation of myocardial apoptosis by terminal deoxynucleotidyltransferase-mediated dUTP nick end labeling (TUNEL) staining and caspace-3 activation revealed that downregulation of MICU1 aggregated MI/R-induced cardiomyocytes loss (Figure 2c). These results indicate that MICU1 is a cardioprotective molecule against MI/R injury, whose mitochondrial expression is inhibited following MI/R.

### MICU1 inhibited mitochondrial Ca^2+^ overload and protected against mitochondrial morphological and functional impairment

**Figure 3:**
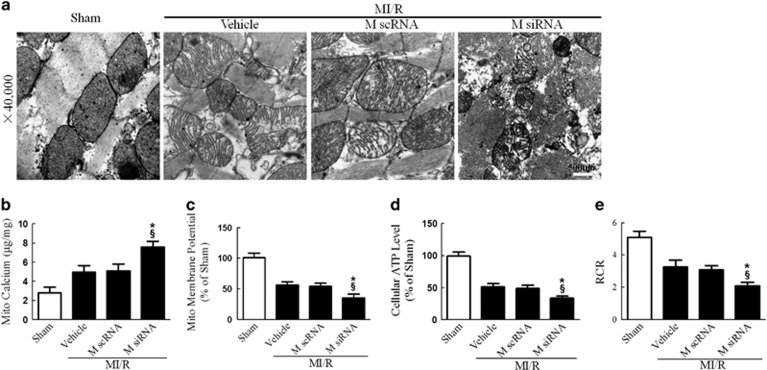
MICU1 deficiency aggravated MI/R-induced mitochondrial injury. (**a**) Representative mitochondrial morphologies detected by transmission electron microscopy. (**b**) Mitochondrial Ca^2+^ content was determined by atomic absorption flame spectroscopy (*μ*g/mg). (**c**) Mitochondrial membrane potential was evaluated using JC-1 kit. (**d**) The ATP content of the myocardium was measured using a firefly luciferase-based ATP assay kit. (**e**) The respiratory control ratio (RCR) was assessed as the ratio of the state 3 to state 4 respiration. Mito, mitochondria; M scRNA, scrambled siRNA used as control; M siRNA, MICU1-specific siRNA. Presented values are means±S.E.M. *N*=6–8/group. *P<0.05 *versus* vehicle of MI/R; ^§^*P*<0.05 *versus* M scRNA of MI/R

The disruption of mitochondrial Ca^2+^ homeostasis is a key event in I/R-induced cellular damage.^3, 7^ Ca^2+^ overload into the mitochondria results in depolarization of the inner mitochondrial membrane and suppression of mitochondrial energetics.^21^ We thus explored the effect of MICU1 on mitochondrial morphology and function. Consistent with previous studies, we found that mitochondrial Ca^2+^ content (assessed by atomic absorption flame spectroscopy) significantly increased in MI/R. More importantly, MICU1 deficiency further aggravated mitochondrial Ca^2+^ overload, destructed mitochondrial morphology (evidenced by disappeared mitochondrial membranes integrity, unusual vesicle-like structures, completely unstructured cristae and ambiguous myofilaments), depressed ATP production and disturbed mitochondrial respiration (Figure 3). These results indicate that MICU1 inhibits post-MI mitochondrial Ca^2+^ overload, promoting structural integrity and subsequently metabolic function.

### Mitochondrial MICU1 content is controlled by the importer receptor Tom70

**Figure 4:**
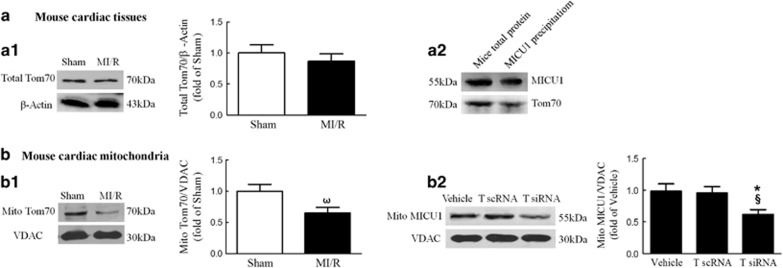
Mitochondrial MICU1 content was controlled by the importer receptor Tom70. (**a1**) Representative western blot images of total Tom70 and *β*-actin in the left panel; intensities of total Tom70 relative to *β*-actin in the right panel. (**a2**) Western blot analysis for MICU1 and Tom70 was performed on the precipitation of MICU1. (**b1**, **b2**) Representative western blot images showing mitochondrial Tom70 or MICU1 expression using VDAC as loading control in the left panel; intensities of mitochondrial Tom70 or MICU1 relative to VDAC in the right panel. Mito, mitochondria; T scRNA, scrambled siRNA used as control; T siRNA, Tom70-specific siRNA; VADC, voltage-dependent anion channel. Presented values are means±S.E.M. *N*=6–8/group. ^ω^*P*<0.05 *versus* sham; *P<0.05 *versus* vehicle; ^§^*P*<0.05 *versus* T scRNA

Our results presented in Figure 1 demonstrated that mitochondrial, but not total, MICU1 is inhibited in cardiac tissue subjected to MI/R. To determine the mechanisms leading to selective inhibition of mitochondrial MICU1 level, the expression level of Tom70, a molecule responsible for initial recognition of mitochondrial pre-proteins from the cytosol,^22^ was determined in I/R heart. As illustrated in Figures 4a1 and b1, Tom70 expression, especially in cardiac mitochondria, was significantly inhibited following 30 min ischemia/3 h reperfusion. Notably, the reduction of mitochondrial Tom70 expression was found as early as 30 min reperfusion, while MICU1 was also proved to decrease as early as 1 h reperfusion (Supplementary Figure 3). To determine whether reduced Tom70 expression is responsible for decreased mitochondrial MICU1 expression, Tom70 expression was inhibited or elevated via intramyocardial injection of siRNA or Tom70-expressing lentivirus (Supplementary Figures 4 and 5). We did not find any changes in basal physiological parameters in both kinds of mice (Table 1). As illustrated in Figure 4b2, downregulation of Tom70 significantly reduced mitochondrial MICU1 content, evidenced by western blot. Moreover, during importing the inner membrane carrier proteins, it is an essential step that mitochondrial precursor proteins require initial recognition by Tom70 to promote the translocation.^23^ Thus, we investigated whether precursor MICU1 coordinated interaction with Tom70 by co-immunoprecipitation. As summarized in Figure 4a2, Tom70 successfully recognized precursor MICU1 in cardiac tissues. These results suggest that mitochondrial MICU1 localization is controlled by the importer receptor Tom70.

### Tom70 deficiency accelerated MI/R injury

**Figure 5:**
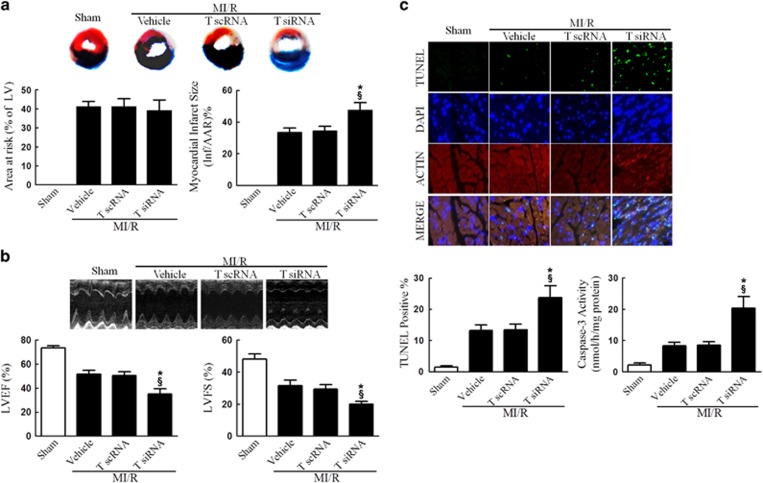
Tom70 deficiency worsened MI/R injury. (**a**) Myocardial infarct size assessed by Evans blue/TTC double staining. (**b**) Cardiac function was assessed by echocardiography. (**c**) Myocardial apoptosis was determined by TUNEL staining and caspase-3 activity assay. T scRNA, scrambled siRNA used as control; T siRNA, Tom70-specific siRNA. Presented values are means±S.E.M. *N*=6–8/group. *P<0.05 *versus* vehicle of MI/R; ^§^*P*<0.05 *versus* T scRNA of MI/R

**Figure 6:**
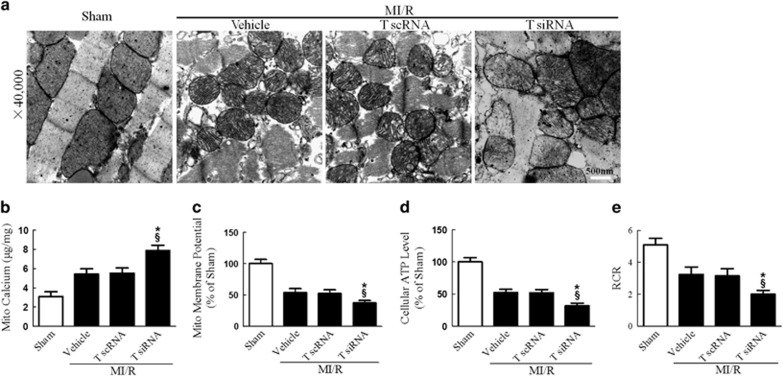
Tom70 deficiency aggravated MI/R-induced mitochondrial injury. (**a**) Representative mitochondrial morphologies detected by transmission electron microscopy. (**b**) Mitochondrial Ca^2+^ content was determined by atomic absorption flame spectroscopy (*μ*g/mg). (**c**) Mitochondrial membrane potential evaluated by JC-1 kit. (**d**) The ATP content measured by ATP assay kit. (**e**) The respiratory control ratio (RCR) was determined as the ratio of the state 3 to state 4 respiration. T scRNA, scrambled siRNA used as control; T siRNA, Tom70-specific siRNA. Presented values are means±S.E.M. *N*=6–8/group. *P<0.05 *versus* vehicle of MI/R; ^§^*P*<0.05 *versus* T scRNA of MI/R

To further identify the role of Tom70 in MI/R injury, we investigated the effects of downregulation of Tom70 on cardiac injuries following I/R. We observed enhanced cTnI leak, enlarged MI size, suppressed cardiac function and increased myocardial apoptosis in Tom70-deficiency mice following MI/R (Figure 5 and Supplementary Figure 6). Moreover, we found that Tom70 deficiency deteriorated MI/R-induced destruction of mitochondrial morphology, depression of mitochondrial function and aggravation of mitochondrial Ca^2+^ overload (Figure 6). These results provide more evidences that Tom70 is a cardioprotective molecule against MI/R injury.

### Tom70 overexpression markedly attenuated MI/R injury, which was partly abolished by MICU1 knockdown

**Figure 7:**
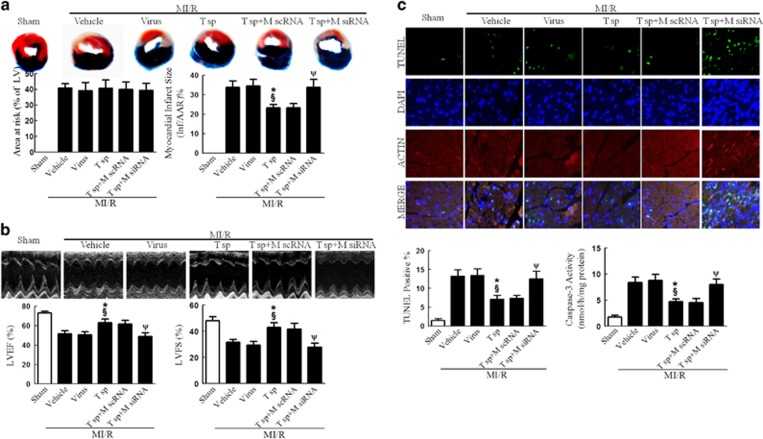
Tom70 supplementation attenuated MI/R injury, which was abolished by MICU1 ablation. (**a**) Myocardial infarct size assessed by Evans blue/TTC double staining. (**b**) Cardiac function was assessed by echocardiography. (**c**) Myocardial apoptosis was determined by TUNEL staining and caspase-3 activity assay. Mito, mitochondria; M scRNA, scrambled siRNA used as control; M siRNA, MICU1-specific siRNA; T sp, Tom70 supplementation; virus, lentivirus vector. Presented values are means±S.E.M. *N*=6–8/group. *P<0.05 *versus* vehicle of MI/R; ^§^*P*<0.05 *versus* virus of MI/R; ^ψ^*P*<0.05 *versus* (T sp+M scRNA) of MI/R

**Figure 8:**
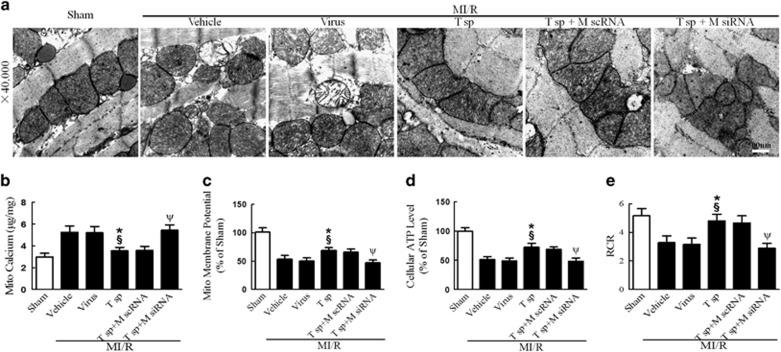
Tom70 supplementation attenuated MI/R-induced mitochondrial injury, which was reversed by MICU1 ablation. (**a**) Representative mitochondrial morphologies detected by transmission electron microscopy. (**b**) Mitochondrial Ca^2+^ content was determined by atomic absorption flame spectroscopy (*μ*g/mg). (**c**) Mitochondrial membrane potential evaluated by JC-1 kit. (**d**) The ATP content measured by ATP assay kit. (**e**) The respiratory control ratio (RCR) was evaluated as the ratio of the state 3 to state 4 respiration. Mito, mitochondria; M scRNA, scrambled siRNA used as control; M siRNA, MICU1-specific siRNA; T sp, Tom70 supplementation; virus, lentivirus vector. Presented values are means±S.E.M. *N*=6–8/group.*P<0.05 *versus* vehicle of MI/R; ^§^*P*<0.05 *versus* virus of MI/R; ^ψ^*P*<0.05 *versus* (T sp+M scRNA) of MI/R

To further determine the causal relationship between MICU1 and Tom70 in mitochondrial function and post-MI cardiac injury, we observed the functional consequence of Tom70 upregulation in MI/R and the involvement of MICU1. As shown in Figures 7 and 8 and Supplementary Figure 7, upregulation of Tom70 significantly improved cardiac function, reduced MI size, suppressed cTnI leak, decreased myocardial apoptosis, preserved mitochondrial morphology and improved mitochondrial function. Mechanistically, Tom70 supplementation successfully prevented mitochondrial Ca^2+^ overload (Figure 8b). However, the Tom70-mediated protection was largely abolished by MICU1 knockdown via intramyocardial injection of siRNA (Figures 7 and 8 and Supplementary Figure 7). These results reveal that MICU1 serves as an indispensable molecule in the protection of Tom70 against MI/R injury. Moreover, the protection provided by MICU1 overexpression was eliminated by Tom70 ablation (Supplementary Figure 8). These results indicate that MICU1 has protective function depending on Tom70 in MI/R.

## Discussion

In the present study, we have made several novel observations. First, we provided the first evidence that although the total expression level of MICU1, a newly identified inhibitory regulator of MCU, was unaltered; its mitochondrial localization was significantly disrupted in ischemic/reperfused heart. Second, we demonstrated that MICU1 exerted protection against MI/R injury via prevention of mitochondrial Ca^2+^ overload, preservation of mitochondrial integrity and promotion of mitochondrial energy metabolism. Third, we found that the expression level of Tom70, the importer receptor having essential role in MICU1 mitochondrial localization, was downregulated after MI/R. Genetic manipulation revealed that loss of Tom70 decreased mitochondrial MICU1 localization and aggravated MI/R injury; whereas, overexpression of Tom70 protected MI/R injury, which was eliminated by MICU1 ablation. Taken together, we have identified a novel cardioprotective pathway involving Tom70/MICU1 signaling, which represents a key regulatory machinery of mitochondrial Ca^2+^ homeostasis by the mitochondrial outer membrane protein transport system (Supplementary Figure 9).

As the cellular ‘powerhouse’ and ‘apoptosis headquarter’, mitochondria have critical roles in various physiological and pathological processes.^24^ As maintenance of Ca^2+^ gradients between cellular compartments depends on ATP-driven reactions, metabolic disruption by injurious stresses, such as MI/R, quickly perturbs cellular Ca^2+^ homeostasis. In particular, release of Ca^2+^ from the endoplasmic reticulum may flood the cytosol with free Ca^2+^, leading to dysfunction of other organelles, particularly mitochondria.^25^ Dysregulation of Ca^2+^ homeostasis has long been implicated to predispose cell injury. Frey *et al.*^26^ revealed that Ca^2+^ overload led to cardiomyocyte death. In response to Ca^2+^ overload, both apoptosis and necrosis can contribute to cardiomyocyte loss by activating pro-death members of the Bcl2 family and opening the mPTP, respectively.^27^ An increase in mitochondrial Ca^2+^ content results in depolarization of the inner mitochondrial membrane, the production of reactive oxygen species and opening of the mPTP, a nonspecific pore in the inner mitochondrial membrane that is permeable to small molecules.

Mitochondrial Ca^2+^ uptake is mediated by the Ca^2+^ uniporter complex in the inner mitochondrial membrane termed MCU, a Ca^2+^-selective ion channel.^14^ MICU1 was recently identified as a protein that localized to the inner mitochondrial membrane and suggested to be required for mitochondrial Ca^2+^ uptake regulation.^13^ MICU1 and MCU biochemically interact, being co-expressed across tissues and species.^11, 15^ Mallilankaraman *et al.*^16^ found that MICU1 was required to preserve normal mitochondrial Ca^2+^ under basal conditions, and served as an essential inhibitory gatekeeper for MCU-mediated mitochondrial Ca^2+^ uptake that regulated cell survival. In our study we found that mitochondrial MICU1 expression was inhibited by MI/R, and MICU1 deficiency deteriorated mitochondrial Ca^2+^ overload and aggravated MI/R injury.

Strong evidences reveal that the Tom complex has critical role in the initial recognition of mitochondrial pre-proteins from the cytosol where the majority of mitochondrial proteins are synthesized and then transported into corresponding compartments in mitochondria.^22^ Many mitochondrial pre-proteins are targeted post-translationally,^28^ and requires initial recognition by import receptors.^18, 29^ Tom70 contains a core region,^28^ within which four C-terminal tetratricopeptide repeat motifs lie to recognize the internal targeting signals of polytopic membrane proteins.^30^ Moreover, Hsp70/Hsp90 chaperones have been reported to integrate the outer membrane-localized Tom70 receptor with mitochondrial preprotein targeting and translocation.^28^ Recently, Li *et al.*^31^ revealed that Tom70 served as an importer receptor to govern the mitochondrial translocation of Opa1 and subsequently exhibited protective effects against pathological cardiac hypertrophy. What about the effect of Tom70 in the mitochondrial translocation of MICU1, particularly the change under MI/R stress? In our study, we found a significant reduction of mitochondrial Tom70 expression since 30 min reperfusion following 30 min ischemia, along with which mitochondrial MICU1 markedly decreased. Consistent with our results, Boengler *et al.*^32^ found that another import receptor of Tom complex Tom20 obviously decreased even after 10 min ischemia/15 min reperfusion. And then, we established Tom70 deficiency and supplementation mice models. As a result, we revealed that MI/R decreased Tom70 expression. Following Tom70 knockdown and supplementation, MI/R injury was aggravated or attenuated, respectively. These results indicate that altered Tom70 expression contributes to MI/R injury. Mechanistically, we demonstrated Tom70 deficiency decreased mitochondrial MICU1 localization and increased Ca^2+^ content. The protection provided by MICU1 overexpression was also eliminated by Tom70 ablation. These results indicate that mitochondrial MICU1 localization and function is restricted by Tom70. Moreover, Tom70 supplementation actually prevented mitochondrial Ca^2+^ overload, in which many factors regulated by Tom70 might have a role. The limitation of our study was hard to determine a specific effector for Tom70 supplementation, we guessed that MICU1 might participate in it. Utilizing cardiac tissues and co-immunoprecipitation, we found that Tom70 interacted with MICU1 in hearts. Thus, the mitochondrial translocation of MICU1 may be governed or facilitated by Tom70 in myocardium. More importantly, MICU1 ablation obviously eliminated the cardioprotection induced by Tom70 overexpression, revealing that MICU1 served as an essential factor to preserve Tom70’s normal function in heart. Consistent with our results, Kato *et al.*^19^ found that Tom70 was essential for PINK1 import into mitochondria, which was reported to decrease the heart’s vulnerability to I/R injury.^20^ Here we revealed MICU1 as a new mitochondrial molecule participating in MI/R. Further studies elucidating the detailed mechanism of mitochondrial translocation of MICU1 by Tom70 receptor is currently undertaking.

## Materials and methods

### Experimental protocols

#### Animals

C57BL6/J mice at the age of 10–12 weeks were used for the present study. All experiments were performed in adherence with the National institutes of Health Guidelines on the Use of Laboratory Animals, and were approved by the Fourth Military Medical University Committee on Animal Care. Mice were fed with standard laboratory animal chow with free access to tap water, and housed in a temperature- and humidity-controlled room with a 12/12 h light–dark cycle.

#### Reagent preparation

Lentivirus vectors were created as previously described.^33^ The *Tom70a* gene-coding sequence was amplified by PCR and subcloned into a lentivirus (LV4) expression plasmid V4886-2 vector to construct a lentivirus-based overexpression vector carrying the *Tom70a* sequence (V4886-2-LV4-*Tom70a*), confirmed by PCR and DNA sequencing (GenePharma, Shanghai, China). Lentivirus expression plasmids were transfected into 293T cells to construct pLenti-GDI viral stock. The titers of the viral vectors used in this study were 1 × 10^9^ TU/ml. In additionally, the siRNAs against MICU1 and Tom70a were designed based on GenBank and purchased from GenePharma. MICU1-specific siRNA (against murine) contains four independent sequences: (1) sense 5′-GCUGGAGCAUCUCUUGAUATT-3′ and antisense 5′-UAUCAAGAGAUGCUCCAGCTT-3′ (2) sense 5′-CUCCACUCCUCAGAGAAAUTT-3′ and antisense 5′-AUUUCUCUGAGGAGUGGAGTT-3′ (3) sense 5′-UUGCCACCUUGAAAGUAAUTT-3′ and antisense 5′-AUUACUUUCAAGGUGGCAATT-3′ and (4) sense 5′-AGCCUUAUCCUGAGGACAATT-3′ and antisense 5′-UUGUCCUCAGGAUAAGGCUTT-3′. Tom70a-specific siRNA (against murine) contains four independent sequences: (1) sense 5′-CAGGCAUAUACAGCAAACATT-3′ and antisense 5′-UGUUUGCUGUAUAUGCCUGTT-3′ (2) sense 5′-CCAGGCAUUAACAGAUCAATT-3′ and antisense 5′-UUGAUCUGUUAAUGCCUGGTT-3′ (3) sense 5′-UGCUGUGUGUAUAUUAGAATT-3′ and antisense 5′-UUCUAAUAUACACACAGCATT-3′ and (4) sense 5′-UGAGAAGAAUGUAGACCUUTT-3′ and antisense 5′-AAGGUCUACAUUCUUCUCATT-3′. Both siRNAs against MICU1 and Tom70a were cotransfected into 293T cells to confirm that those designed sequences possessing knockdown efficiency, respectively.

### *In vivo* delivery of siRNA and lentivirus of MICU1 and Tom70

A unit of 20 *μ*g of MICU1 siRNA, Tom70 siRNA or scrambled siRNA was diluted in 30 *μ*l of vivo-jetPEI^TM^ (Invitrogen, Carlsbad, CA, USA) and 10% glucose mixture for preparation. Animals were first anesthetized with 2% isoflurane, and an incision was made between the fourth and fifth left ribs to expose the heart. A volume of 30 *μ*l of vehicle, scrambled siRNA, MICU1 siRNA or Tom70 siRNA solution were delivered via intramyocardial injection into the apex and anterolateral wall using a 30-gauge needle. Another group of mice were injected with 30 *μ*l of vehicle, 3.3 × 10^7^ TU V4886-2-LV4, 3.3 × 10^7^ TU V4886-2-LV4-*Tom70a*, (3.3 × 10^7^ TU V4886-2-LV4-*Tom70a*+20 *μ*g MICU1 scRNA) or (3.3 × 10^7^ TU V4886-2-LV4-*Tom70a*+20 *μ*g MICU1 siRNA).

### MI/R model

After 72 h of siRNA or lentivirus injection in hearts, the incision was opened under anesthetic condition once again. MI was introduced by temporarily exteriorizing the heart via a left thoracic incision and placing a 6-0 silk suture slipknot around the left anterior descending coronary artery. After 30 min of MI, the slipknot was released, and the myocardium was reperfused for 3 h (for cell apoptosis, biochemical assays and mitochondrial morphology/function assessment) or 24 h (for cardiac function and infarct size determination). Sham-operated control mice (sham MI/R) underwent the same surgical procedures except that the suture placed under the left coronary artery was not tied.^34^

### Measurement of cardiac function and myocardial infarct size

At the end of 24 h reperfusion, mice were re-anesthetized with isoflurane. Left ventricular ejection fraction and fractioning shortening were obtained by transthoracic echocardiography. After functional determination, the ligature around the coronary artery was retied, and myocardial infarct size was determined by the Evans blue/TTC (Sigma, St Louis, MO, USA) double-staining method, as described previously.^35^

### Determination of cTnI

After 30 min of ischemia and 3 h of reperfusion, mice were completely exsanguinated. The circulating levels of specific isoform of cTnI were tested with an ELISA kit following the manufacturer’s instructions (Life Diagnostics, West Chester, PA, USA).^36^

### Determination of myocardial apoptosis

Myocardial apoptosis was determined by TUNEL staining (Roche, Nutley, NJ, USA). The paraffin-embedded tissue was cut into 4–5 *μ*m-thick sections. The sections were then incubated in 50 *μ*l of the TUNEL mixture (47.5 *μ*l of TUNEL label containing fluorescein isothiocyanate-conjugated dUTP and 2.5 *μ*l of TUNEL enzyme) in a humidified chamber (60 min, 37 °C). Control sections were incubated with 50 *μ*l of TUNEL label solution containing no TUNEL enzyme. An additional staining was performed with monoclonal anti-*α*-sarcomeric actin (Sigma). All sections were photographed with a QICAM-Fast Digital Camera mounted atop an Olympus BX51 Fluorescence Microscope (Olympus America Inc, Center Valley, PA, USA). Total nuclei and the TUNEL-positive nuclei were counted by IP Lab Imaging Analysis Software (Version 3.5; Scanalytics, Fairfax, VA, USA). Apoptotic index (number of TUNEL-positive nuclei/total number of nuclei × 100) was automatically calculated and exported to Microsoft Excel for further analysis. Cardiac caspase-3 activity was performed by using a caspase-3 colorimetric assay kit (Keygen, Nanjing, China), following the manufacturer’s instructions. Myocardial tissues were homogenized in ice-cold lysis buffer for 30 s. The homogenates were centrifuged (10 000 × *g* × 5 min, 4 °C) and the supernatants were collected. Supernatant containing 200 *μ*g of protein was loaded to each well of the 96-well plate and incubated with 25 *μ*g Ac-DEVD-pNA at 37 °C for 1.5 h. The pNA absorbance was quantified using a SpectraMax plate reader (SpectraMax, Atlanta, GA, USA) at 405 nm.^37^

### Isolation of fresh cardiac mitochondria

Cardiac tissues of IR region (area at risk, which was controlled by left anterior descending coronary artery, that is, the apex and anterolateral wall of the heart) were minced in mitochondria isolation buffer (250 mM sucrose, 10 mM HEPES, 1 mM EGTA and 0.5% BSA, pH 7.4). The tissue was buffer-washed several times to remove blood and homogenized (Potter–Elvehjem). Subsequently, the lysates were centrifuged at 700 × *g* for 10 min. The supernatant was filtered through a nylon filter of 250 nm and then centrifuged at 10 780 × *g* for 10 min. The pellet was re-suspended in mitochondria isolation buffer and centrifuged at 7650 × *g* for 10 min. Again, the pellet was re-suspended in mitochondria isolation buffer, then layered on top of a 30% percoll solution in isolation buffer and centrifuged at 35 000 × *g* for 30 min. The mitochondria was collected and washed twice in mitochondria isolation buffer by centrifugation at 7650 × *g* for 5 min.^32^

### Immunoblotting

Cardiac tissues of IR region were lysed, sonicated and centrifuged. Mitochondria isolated from fresh cardiac tissues were lysed with lysis buffer (25 mM Tris-HCl, pH 7.6, 150 mM NaCl, 2 mM EDTA, 1% sodium deoxycholate, 0.5% SDS, 1% Triton X-100 and protease inhibitor). After sonication, the lysates were centrifuged at 12 000 × *g* for 30 min to remove residue. Protein concentrations were measured using Bio-Rad kit (Bio-Rad Laboratories, Hercules, CA, USA). Proteins were separated by SDS-PAGE and then transferred to a polyvinylidenedifluoride membrane (Millipore, Bedford, MA, USA). After blocking with 5% skim milk in 1 × PBST at room temperature for 1 h, the membrane were incubated with primary antibody against MICU1 (1:500; Immunoway, CA, USA), Tom70 (1:500; Santa Cruz Biotechnology, Santa Cruz, CA, USA), *β*-actin (1:3000; Sigma) or VDAC (1:3000; Sigma) overnight at 4 °C. The membrane was then washed with 1 × PBST and incubated with horseradish peroxidase-conjugated IgG antibody (Cell Signaling, Beverly, MA, USA) for 1 h at room temperature. The blots were visualized using a super signal chemiluminescence detection kit (Thermo Scientific, Waltham, MA, USA) and exposure to X-ray film.^38^

### Immunohistochemistry and immunofluorescence staining

Myocardial tissues were fixed with 4% paraformaldehyde, and paraffin sections (3–5 *μ*m) were prepared for immunohistochemistry and immunofluorescence staining. For immunohistochemistry, the sections were blocked with 10% normal goat serum for 1 h and probed with anti-Tom70 antibody (1:20, Santa Cruz) at 4 °C overnight. Then they were incubated with biotinylated goat anti-mouse IgG at room temperature for 2 h and incubated with ExtrAvidin Peroxidase (Sigma/Aldrich Quimica, Madrid, Spain) for 1 h. Positive staining was detected with 0.05% DAB/0.01% H_2_O_2_ in 0.05 mol/l Tris-HCl buffer. The negative control group, in which one of the primary antibodies was omitted and replaced with normal IgG, showed no immunoreactivity. Nuclei were counterstained with 2-(4-amidino-phenyl)-6-indolecarba-midine dihydrochloride (DAPI). All photographs were taken under an Olympus BX51 Fluorescence Microscope (Olympus America Inc).

The spatial expression of MICU1 was evaluated with immunofluorescence using anti-MICU1 antibody at 4 °C overnight. All labeled sections were incubated with antibodies against MICU1 (1:50; Immunoway) in a humidified container at 4 °C overnight. After washing with PBS, the sections were incubated with tetramethylrhodamineisothiocyanate-conjugated second antibodies. After washing three times with PBS, DAPI (Beyotime, Shanghai, China) solution was added to stain the cell nucleus for 3 min. Sections were then washed in PBS and sealed with a coverslip. The slides were analyzed with laser confocal microscopy with excitation at 488 nm (Olympus, FV1000). Image J software (National Institutes of Health, Bethesda, MD, USA) was used for quantitative analysis, and total antibody staining was normalized to DAPI.^39^

### Transmission electron microscopy

Hearts were removed and flushed in ice-cold PBS. Left ventricular walls were cut perpendicular to the long axis and trimmed into 1–2 mm-wide blocks. After fixing overnight in 4% glutaraldehyde, the sections were postfixed in 1% osmium tetroxide for 1 h, dehydrated using a graded ethanol immersion series and embedded in resin. Tissue pieces were cut into 80 nm-thick sections by ultramicrotome (Leica, Vienna, Austria). The ultrathin sections were fixed on 200-slot grids coated with piloform membrane and observed with a JEM-1400 electron microscope (JEOL, Tokyo, Japan) and the micrographs were captured with CCD camera (OLYMPUE, Tokyo, Japan). Mitochondria were imaged at 40 000 (40K) magnifications, and representative images were acquired as described previously.^40^

### Evaluation of mitochondrial respiration

Mitochondrial respiration was tested at 25 °C by using a Clark-type electrode connected to a respiration chamber (YSI Incorporated, Yellow Springs, OH, USA) and a linear chart recorder, in buffer (pH 7.4) containing 20 mM HEPES, 10 mM KCl, 5 mM KH2PO4, 2.5 mM MgCl2, 0.25 M sucrose, 0.2 mM EDTA and 1 mg/ml fatty acid-free BSA. Intact cardiac mitochondria were added to 0.5 mg protein per ml. For glutamate/malate respiration evaluation, substrates were added each at 5 mM. With the addition of 0.5 mM ADP, state 3 respiration was tested. In the absence of ADP, state 4 respiration was determined. The respiratory control ratio was calculated as the ratio of the state 3 to state 4 respiration.^41^

### Assessment of ATP content

The ATP content of the myocardium was measured using a firefly luciferase-based ATP assay kit (Beyotime) according to the manufacturer’s instructions. Cardiac tissues were homogenized and centrifuged at 12 000 × *g* for 5 min. Supernatants were mixed with ATP detection working dilution in a white 96-well plate. Luminance (RLU) was measured by using an Infinite M200 Microplate Reader (LabX, Midland, ON, Canada). Standard curves were also generated and the protein concentration of each treatment group was determined using the Bradford protein assay. Total ATP levels were expressed as nmol/mg protein.^42^

### Measurement of mitochondrial membrane potential (ΔΨm)

The quantification of mitochondrial potential variation was evaluated using JC-1 (Sigma, Taufkirchen, Germany). Cardiomyocytes (≈50 cells from 8 mice per group) isolated from mice were seeded on gelatin-coated culture chamber slides and stained with JC-1 (5 *μ*mol/l) at 37 °C for 10 min. Cells were rinsed with the HEPES-saline buffer. Fluorescence of each sample was read at excitation wavelength of 490 nm and emission wavelength of 530 and 590 nm using a spectrofluorimeter (SpectraMax Gemini XS, SpectraMax). In healthy cells, a high concentration of JC-1 forms aggregates that yield red fluorescence at ≈590 nm. In unhealthy cells, JC-1 exists as a monomer at low concentration emitting red fluorescence at ≈530 nm. Results in fluorescence intensity were expressed as the ratio of 590 to 530 nm emission.^43^

### Determination of mitochondrial Ca^2+^ content by atomic absorption flame spectroscopy

Mitochondrial samples for atomic absorption for quantification of calcium were dried at 200 °C in an oven for 2 h, weighed in its entirety and hydrolyzed in glass tubes containing 1 ml of 6 M hydrochloric acid. The tubes were heated to 92 °C in a water bath. The hydrolyzed samples were then sent for spectrometry analysis (PERKIN ELMER 4100, LACTEC-PR, Brazil), and the results were expressed in microgram of calcium per microgram of tissue mitochondrial proteins.^44^

### Immunoprecipitation of MICU1 with Tom70

MICU1 antibody was bound to pre-washed protein A/G Plus Agarose (Santa Cruz Biotechnology, Santa Cruz, CA, USA). The beads were then blocked with 1 mg BSA and washed once with IP buffer (150 mM NaCl, 1.5 mM MgCl2, 1 mM EGTA and 20 mM HEPES (pH 7.4) buffer containing 1% NP40). The diluted myocardial extracts were added to the beads and rotated at 4 °C for 2 h. The beads were washed three times with IP buffer and extracted with 20 *μ*l of 2 × sample buffer containing 20 mM DTT. The eluates were then subjected to western blot analysis.

### Statistical analysis

All values are expressed as mean±S.E.M. and obtained from six to eight separate experimental preparations. Data (except western blot density) were subjected to ANOVA followed by Bonferroni correction for *post hoc t*-test. Western blot densities were analyzed with the Kruskal–Wallis test followed by Dunn *post hoc* test. *P*<0.05 was considered statistically significant.

## Figures and Tables

**Table 1:** Physiological measurements

**Basal physiological parameters in all kinds of mice**					
	**Control**	**MICU1 KD**	**Tom70 KD**	**Tom70 sp**	**Tom70 sp+MICU1 KD**
HW (mg)	121.0±5.4	120.9±4.7	119.5±5.9	123.5±3.8	126.4± 6.7
BW (g)	24.7±1.2	23.7±2.1	23.9±1.5	24.7±2.3	24.3±1.8
HW/BW (mg/g)	4.9±0.2	5.1±0.4	5.0±0.3	5.0±0.2	5.2±0.5
HR (b.p.m.)	410±36	418±27	421±39	401±23	419±45
LVEF (%)	71±3.8	68±4.6	69±3.5	70±2.9	68±4.1
LVFS (%)	43±2.3	39±3.6	40±4.2	41±2.7	39±3.5

Abbreviations: BW, body weight; HR, heart rate; HW, heart weight; HW/BW, heart weight/body weight ratio; KD, knockdown; LVEF, left ventricle ejection fraction; LVFS, left ventricle fractional shortening; sp, supplementation

Cardiac weight index was defined as the ratio of heart weight/body weight (in mg/g). Cardiac function was assessed in anesthetized mice by transthoracic echocardiography

## References

[bib1] Zhang Y, Ren J. Targeting autophagy for the therapeutic application of histone deacetylase inhibitors in ischemia/reperfusion heart injury. Circulation 2014; 129: 1088–1091. 24396040 10.1161/CIRCULATIONAHA.113.008115

[bib2] Xie M, Kong Y, Tan W, May H, Battiprolu PK, Pedrozo Z et al. Histone deacetylase inhibition blunts ischemia/reperfusion injury by inducing cardiomyocyte autophagy. Circulation 2014; 129: 1139–1151. 24396039 10.1161/CIRCULATIONAHA.113.002416 PMC3984913

[bib3] Murphy E, Steenbergen C. Mechanisms underlying acute protection from cardiac ischemia-reperfusion injury. Physiol Rev 2008; 88: 581–609. 18391174 10.1152/physrev.00024.2007 PMC3199571

[bib4] Solaini G, Harris DA. Biochemical dysfunction in heart mitochondria exposed to ischaemia and reperfusion. Biochem J 2005; 390: 377–394. 16108756 10.1042/BJ20042006 PMC1198918

[bib5] Kubli DA, Gustafsson AB. Mitochondria and mitophagy: the yin and yang of cell death control. Circ Res 2012; 111: 1208–1221. 23065344 10.1161/CIRCRESAHA.112.265819 PMC3538875

[bib6] Balaban RS. The role of Ca(2+) signaling in the coordination of mitochondrial ATP production with cardiac work. Biochim Biophys Acta 2009; 1787: 1334–1341. 19481532 10.1016/j.bbabio.2009.05.011 PMC3177847

[bib7] Aurora AB, Mahmoud AI, Luo X, Johnson BA, van Rooij E, Matsuzaki S et al. MicroRNA-214 protects the mouse heart from ischemic injury by controlling Ca(2)(+) overload and cell death. J Clin Invest 2012; 122: 1222–1232. 22426211 10.1172/JCI59327 PMC3314458

[bib8] Liu T, Takimoto E, Dimaano VL, DeMazumder D, Kettlewell S, Smith G et al. Inhibiting mitochondrial Na+/Ca2+ exchange prevents sudden death in a Guinea pig model of heart failure. Circ Res 2014; 115: 44–54. 24780171 10.1161/CIRCRESAHA.115.303062 PMC4219273

[bib9] Brookes PS, Yoon Y, Robotham JL, Anders MW, Sheu SS. Calcium, ATP, and ROS: a mitochondrial love-hate triangle. Am J Physiol Cell Physiol 2004; 287: C817–C833. 15355853 10.1152/ajpcell.00139.2004

[bib10] Marchi S, Pinton P. The mitochondrial calcium uniporter complex: molecular components, structure and physiopathological implications. J Physiol 2014; 592: 829–839. 24366263 10.1113/jphysiol.2013.268235 PMC3948548

[bib11] De Stefani D, Raffaello A, Teardo E, Szabo I, Rizzuto R. A forty-kilodalton protein of the inner membrane is the mitochondrial calcium uniporter. Nature 2011; 476: 336–340. 21685888 10.1038/nature10230 PMC4141877

[bib12] Luongo TS, Lambert JP, Yuan A, Zhang X, Gross P, Song J et al. The mitochondrial calcium uniporter matches energetic supply with cardiac workload during stress and modulates permeability transition. Cell Rep 2015; 12: 23–34. 26119731 10.1016/j.celrep.2015.06.017 PMC4517182

[bib13] Perocchi F, Gohil VM, Girgis HS, Bao XR, McCombs JE, Palmer AE et al. MICU1 encodes a mitochondrial EF hand protein required for Ca(2+) uptake. Nature 2010; 467: 291–296. 20693986 10.1038/nature09358 PMC2977980

[bib14] Logan CV, Szabadkai G, Sharpe JA, Parry DA, Torelli S, Childs AM et al. Loss-of-function mutations in MICU1 cause a brain and muscle disorder linked to primary alterations in mitochondrial calcium signaling. Nat Genet 2014; 46: 188–193. 24336167 10.1038/ng.2851

[bib15] Baughman JM, Perocchi F, Girgis HS, Plovanich M, Belcher-Timme CA, Sancak Y et al. Integrative genomics identifies MCU as an essential component of the mitochondrial calcium uniporter. Nature 2011; 476: 341–345. 21685886 10.1038/nature10234 PMC3486726

[bib16] Mallilankaraman K, Doonan P, Cardenas C, Chandramoorthy HC, Muller M, Miller R et al. MICU1 is an essential gatekeeper for MCU-mediated mitochondrial Ca(2+) uptake that regulates cell survival. Cell 2012; 151: 630–644. 23101630 10.1016/j.cell.2012.10.011 PMC3486697

[bib17] Baker MJ, Frazier AE, Gulbis JM, Ryan MT. Mitochondrial protein-import machinery: correlating structure with function. Trends Cell Biol 2007; 17: 456–464. 17825565 10.1016/j.tcb.2007.07.010

[bib18] Yamamoto H, Fukui K, Takahashi H, Kitamura S, Shiota T, Terao K et al. Roles of Tom70 in import of presequence-containing mitochondrial proteins. J Biol Chem 2009; 284: 31635–31646. 19767391 10.1074/jbc.M109.041756 PMC2797234

[bib19] Kato H, Lu Q, Rapaport D, Kozjak-Pavlovic V. Tom70 is essential for PINK1 import into mitochondria. PLoS ONE 2013; 8: e58435. 23472196 10.1371/journal.pone.0058435 PMC3589387

[bib20] Siddall HK, Yellon DM, Ong SB, Mukherjee UA, Burke N, Hall AR et al. Loss of PINK1 increases the heart's vulnerability to ischemia-reperfusion injury. PLoS ONE 2013; 8: e62400. 23638067 10.1371/journal.pone.0062400 PMC3639249

[bib21] Weiss JN, Korge P, Honda HM, Ping P. Role of the mitochondrial permeability transition in myocardial disease. Circ Res 2003; 93: 292–301. 12933700 10.1161/01.RES.0000087542.26971.D4

[bib22] Neupert W, Herrmann JM. Translocation of proteins into mitochondria. Annu Rev Biochem 2007; 76: 723–749. 17263664 10.1146/annurev.biochem.76.052705.163409

[bib23] Fan AC, Kozlov G, Hoegl A, Marcellus RC, Wong MJ, Gehring K et al. Interaction between the human mitochondrial import receptors Tom20 and Tom70 *in vitro* suggests a chaperone displacement mechanism. J Biol Chem 2011; 286: 32208–32219. 21771790 10.1074/jbc.M111.280446 PMC3173181

[bib24] Hollander JM, Thapa D, Shepherd DL. Physiological and structural differences in spatially distinct subpopulations of cardiac mitochondria: influence of cardiac pathologies. Am J Physiol Heart Circ Physiol 2014; 307: H1–14. 24778166 10.1152/ajpheart.00747.2013 PMC4080170

[bib25] Orrenius S, McConkey DJ, Bellomo G, Nicotera P. Role of Ca2+ in toxic cell killing. Trends Pharmacol Sci 1989; 10: 281–285. 2672472 10.1016/0165-6147(89)90029-1

[bib26] Frey N, McKinsey TA, Olson EN. Decoding calcium signals involved in cardiac growth and function. Nat Med 2000; 6: 1221–1227. 11062532 10.1038/81321

[bib27] Dorn GW 2nd. Apoptotic and non-apoptotic programmed cardiomyocyte death in ventricular remodelling. Cardiovasc Res 2009; 81: 465–473. 18779231 10.1093/cvr/cvn243 PMC2721651

[bib28] Young JC, Hoogenraad NJ, Hartl FU. Molecular chaperones Hsp90 and Hsp70 deliver preproteins to the mitochondrial import receptor Tom70. Cell 2003; 112: 41–50. 12526792 10.1016/s0092-8674(02)01250-3

[bib29] Yamano K, Yatsukawa Y, Esaki M, Hobbs AE, Jensen RE, Endo T. Tom20 and Tom22 share the common signal recognition pathway in mitochondrial protein import. J Biol Chem 2008; 283: 3799–3807. 18063580 10.1074/jbc.M708339200

[bib30] Lithgow T, Glick BS, Schatz G. The protein import receptor of mitochondria. Trends Biochem Sci 1995; 20: 98–101. 7709435 10.1016/s0968-0004(00)88972-0

[bib31] Li J, Qi M, Li C, Shi D, Zhang D, Xie D et al. Tom70 serves as a molecular switch to determine pathological cardiac hypertrophy. Cell Res 2014; 24: 977–993. 25022898 10.1038/cr.2014.94 PMC4123302

[bib32] Boengler K, Gres P, Cabestrero A, Ruiz-Meana M, Garcia-Dorado D, Heusch G et al. Prevention of the ischemia-induced decrease in mitochondrial Tom20 content by ischemic preconditioning. J Mol Cell Cardiol 2006; 41: 426–430. 16828795 10.1016/j.yjmcc.2006.05.015

[bib33] Kobinger GP, Weiner DJ, Yu QC, Wilson JM. Filovirus-pseudotyped lentiviral vector can efficiently and stably transduce airway epithelia *in vivo*. Nat Biotechnol 2001; 19: 225–230. 11231554 10.1038/85664

[bib34] Gao E, Lei YH, Shang X, Huang ZM, Zuo L, Boucher M et al. A novel and efficient model of coronary artery ligation and myocardial infarction in the mouse. Circ Res 2010; 107: 1445–1453. 20966393 10.1161/CIRCRESAHA.110.223925 PMC3005817

[bib35] Pei H, Yu Q, Xue Q, Guo Y, Sun L, Hong Z et al. Notch1 cardioprotection in myocardial ischemia/reperfusion involves reduction of oxidative/nitrative stress. Basic Res Cardiol 2013; 108: 373. 23989801 10.1007/s00395-013-0373-x

[bib36] Elrod JW, Calvert JW, Morrison J, Doeller JE, Kraus DW, Tao L et al. Hydrogen sulfide attenuates myocardial ischemia-reperfusion injury by preservation of mitochondrial function. Proc Natl Acad Sci USA 2007; 104: 15560–15565. 17878306 10.1073/pnas.0705891104 PMC2000503

[bib37] Pei H, Qu Y, Lu X, Yu Q, Lian K, Liu P et al. Cardiac-derived adiponectin induced by long-term insulin treatment ameliorates myocardial ischemia/reperfusion injury in type 1 diabetic mice via AMPK signaling. Basic Res Cardiol 2013; 108: 322. 23262803 10.1007/s00395-012-0322-0

[bib38] Liu S, Yin T, Wei X, Yi W, Qu Y, Liu Y et al. Downregulation of adiponectin induced by tumor necrosis factor alpha is involved in the aggravation of posttraumatic myocardial ischemia/reperfusion injury. Crit Care Med 2011; 39: 1935–1943. 21499085 10.1097/CCM.0b013e31821b85db

[bib39] Li T, Hu J, He GH, Li Y, Zhu CC, Hou WG et al. Up-regulation of NDRG2 through nuclear factor-kappa B is required for Leydig cell apoptosis in both human and murine infertile testes. Biochim Biophys Acta 2012; 1822: 301–313. 22138128 10.1016/j.bbadis.2011.11.013

[bib40] Liu L, Feng D, Chen G, Chen M, Zheng Q, Song P et al. Mitochondrial outer-membrane protein FUNDC1 mediates hypoxia-induced mitophagy in mammalian cells. Nat Cell Biol 2012; 14: 177–185. 22267086 10.1038/ncb2422

[bib41] Zhou HZ, Ma X, Gray MO, Zhu BQ, Nguyen AP, Baker AJ et al. Transgenic MMP-2 expression induces latent cardiac mitochondrial dysfunction. Biochem Biophys Res Commun 2007; 358: 189–195. 17475219 10.1016/j.bbrc.2007.04.094 PMC3423089

[bib42] Yue R, Hu H, Yiu KH, Luo T, Zhou Z, Xu L et al. Lycopene protects against hypoxia/reoxygenation-induced apoptosis by preventing mitochondrial dysfunction in primary neonatal mouse cardiomyocytes. PLoS ONE 2012; 7: e50778. 23226382 10.1371/journal.pone.0050778 PMC3511264

[bib43] Zhang S, Liu X, Bawa-Khalfe T, Lu LS, Lyu YL, Liu LF et al. Identification of the molecular basis of doxorubicin-induced cardiotoxicity. Nat Med 2012; 18: 1639–1642. 23104132 10.1038/nm.2919

[bib44] Collatusso C, Roderjan JG, Vieira ED, Costa FD, Noronha L, Fornazari Dde F. Effect of SDS-based decelullarization in the prevention of calcification in glutaraldehyde-preserved bovine pericardium: study in rats. Rev Bras Cir Cardiovasc 2012; 27: 88–96. 22729305 10.5935/1678-9741.20120013

