# Supplementary material for: MICU1 protects against myocardial ischemia/reperfusion injury and its control by the importer receptor Tom70
Source: Cell Death Dis. 2017 Jul 13;8(7):e2923–. doi: 10.1038/cddis.2017.280 (PMC5550843; doi:10.1038/cddis.2017.280)

**Supplemental Figure Legends:**

**Supplemental Figure1. Mitochondrial MICU1 knockdown mice were established.** (A) Cardiac MICU1 expression was evaluated by immunofluorescence. (B) Representative western blot images showing mitochondrial MICU1 expression using VDAC as loading control in the upper panel, intensities of MICU1 relative to VDAC in the lower panel. (C) Mitochondrial calcium concentration was tested by atomic absorption flame spectroscopy (μg/mg). (D) The respiratory control ratio (RCR) was assessed as the ratio of the state 3 to state 4 respiration. (E) Myocardial apoptosis was determined by caspase-3 activity assay. (F) Left ventricle ejection fraction (LVEF) was assessed by echocardiography in mice. M siRNA, MICU1 specific siRNA; M scRNA, Scrambled siRNA used as control; VADC, Voltage-dependent anion channel; Mito, Mitochondria. Presented values are means ± SEM. N=6 to 8/group. ^**^*P*<0.01 vs. Vehicle; ^§§^*P*<0.01 vs. M scRNA.


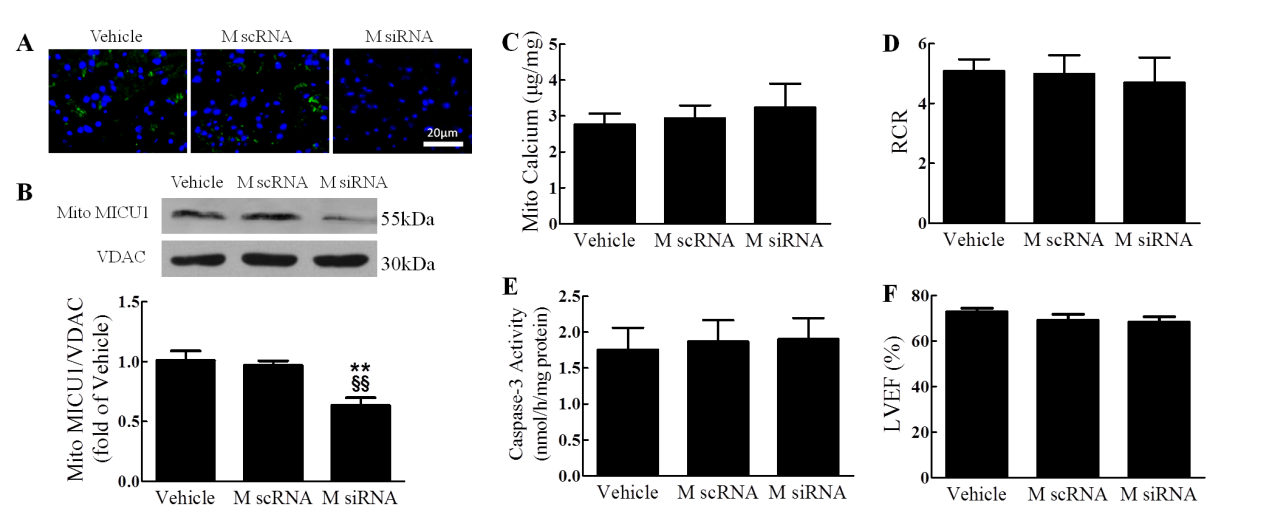


**Supplemental Figure2. MICU1 deficiency aggravated I/R-induced cTnI leak and cardiac function suppression.** (A) After 3 hours of reperfusion, cTnI level was measured by using an ELISA kit. (B,C) Cardiac function was assessed by echocardiography in mice 3 days after MI/R injury. MI/R, myocardial ischemia/reperfusion; I/R, ischemia/reperfusion; M siRNA, MICU1 specific siRNA; M scRNA, Scrambled siRNA used as control; cTnI, cardiac troponin-I; LVEF, Left ventricle ejection fraction; LVFS, Left ventricle fractional shortening. Presented values are means ± SEM. N=6 to 8/group. ^*^*P*<0.05 vs. Vehicle of MI/R; ^§^*P*<0.05 vs. M scRNA of MI/R.

**
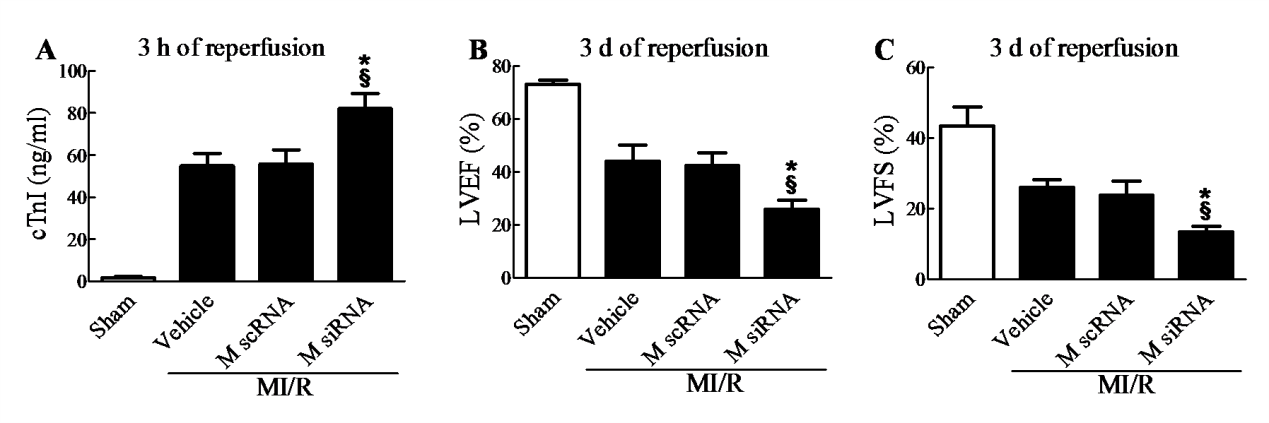
**

**Supplemental Figure3. MI/R significantly decreased mitochondrial expressions of Tom70 and MICU1.** (A) Representative western blot images showing mitochondrial Tom70 expression using VDAC as loading control in the upper panel, intensities of Tom70 relative to VDAC in the lower panel. (B) Representative western blot images showing mitochondrial MICU1 expression using VDAC as loading control in the upper panel, intensities of MICU1 relative to VDAC in the lower panel. MI/R, myocardial ischemia/reperfusion; VADC, Voltage-dependent anion channel; Mito, Mitochondria. Presented values are means ± SEM. N=6 to 8/group. ^*^*P*<0.05 vs. Sham.

**
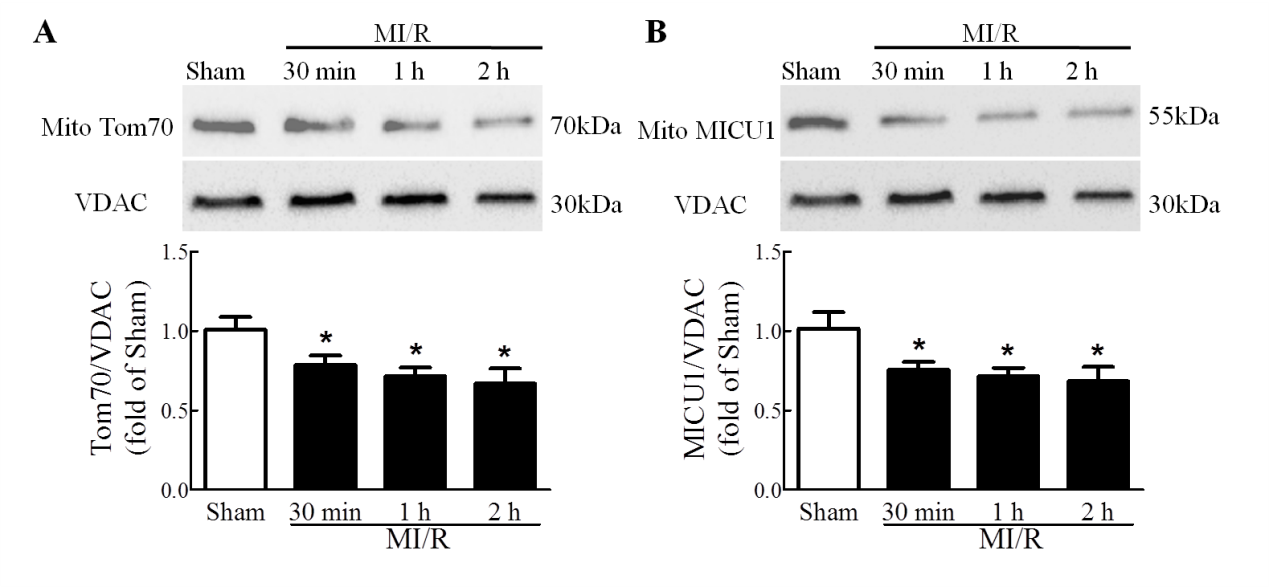
**

**Supplemental Figure4. Mitochondrial Tom70 knockdown mice were established.** (A) Cardiac Tom70 expression was evaluated by immunohistochemistry staining. (B) Representative western blot images showing mitochondrial Tom70 expression using VDAC as loading control in the upper panel, intensities of Tom70 relative to VDAC in the lower panel. (C) Mitochondrial calcium concentration was assessed by atomic absorption flame spectroscopy (μg/mg). (D) The respiratory control ratio (RCR) was calculated as the ratio of the state 3 to state 4 respiration. T siRNA, Tom70 specific siRNA; T scRNA, Scrambled siRNA used as control; VADC, Voltage-dependent anion channel; Mito, Mitochondria. Presented values are means ± SEM. N=6 to 8/group. ^**^*P*<0.01 vs. Vehicle; ^§§^*P*<0.01 vs. T scRNA.


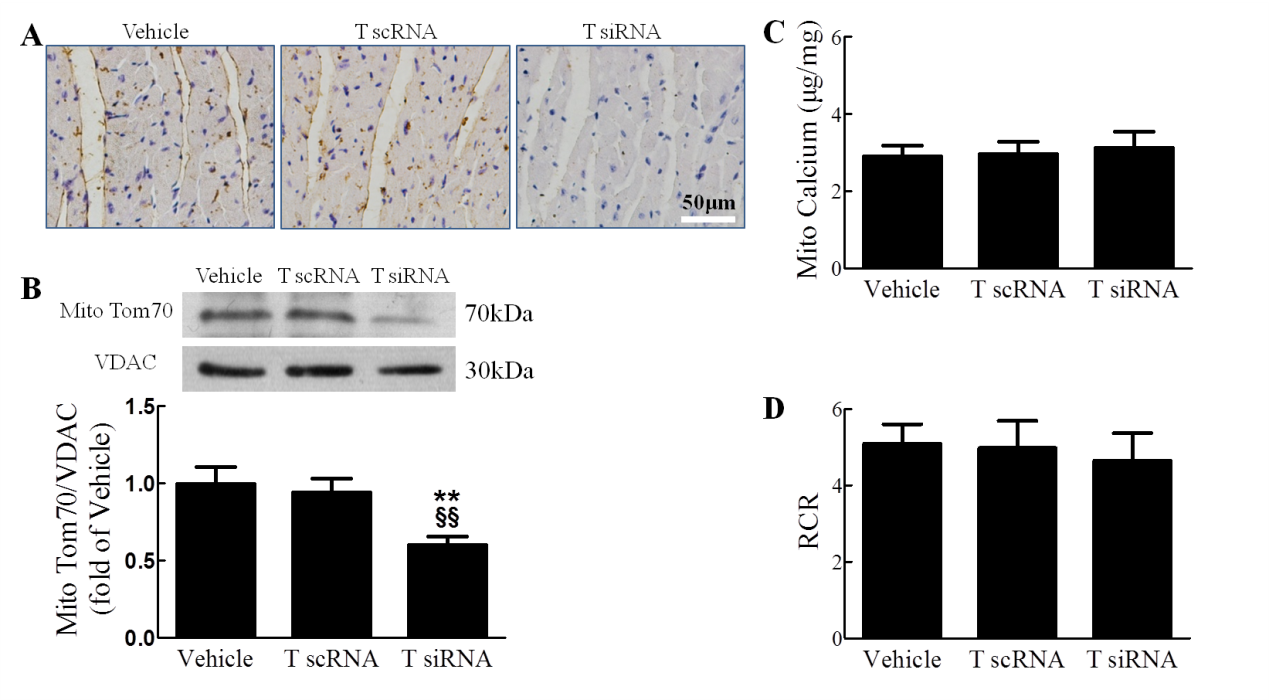


**Supplemental Figure5. Mitochondrial Tom70 supplementation mice were established.** (A) Cardiac Tom70 expression were evaluated by immunohistochemistry staining. (B) Representative western blot images showing mitochondrial Tom70 expression using VDAC as loading control in the upper panel, intensities of Tom70 relative to VDAC in the lower panel. (C) Mitochondrial calcium concentration was measured by atomic absorption flame spectroscopy (μg/mg). (D) The respiratory control ratio (RCR) was determined as the ratio of the state 3 to state 4 respiration. T sp, Tom70 supplementation; VADC, Voltage-dependent anion channel; Mito, Mitochondria. Presented values are means ± SEM. N=6 to 8/group. ^**^*P*<0.01 vs. Vehicle; ^§§^*P*<0.01 vs. Virus.


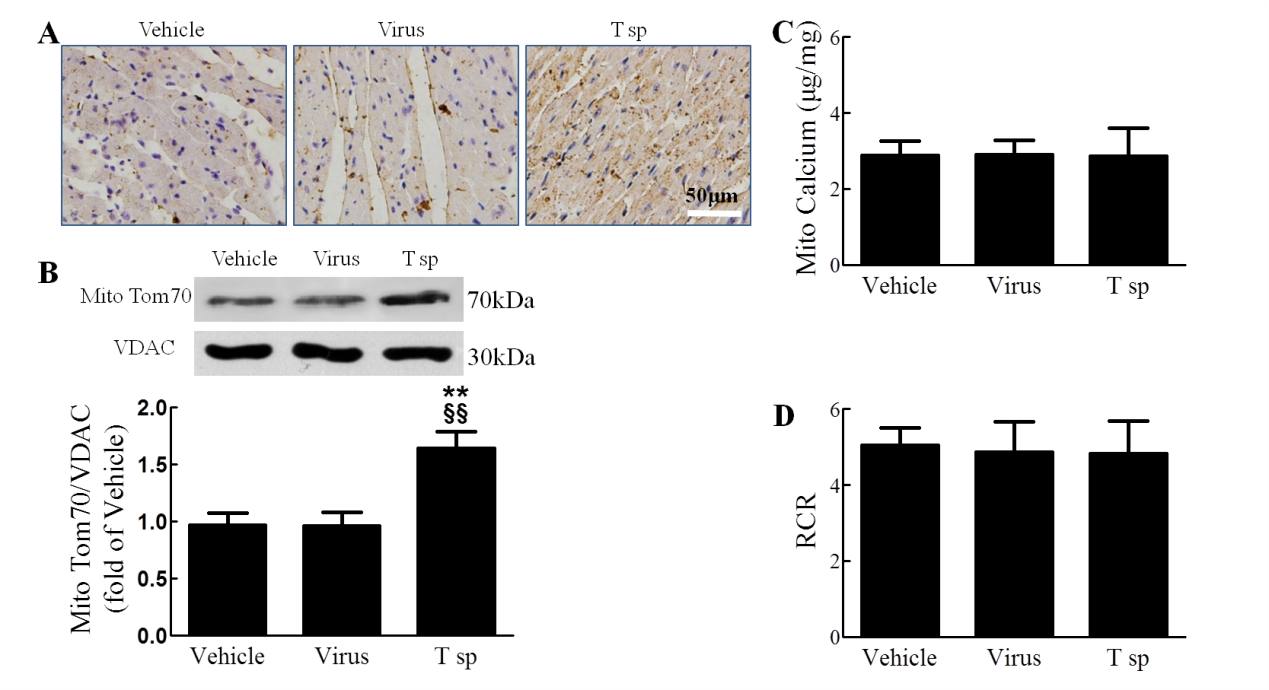


**Supplemental Figure6. Tom70 deficiency aggravated I/R-induced cTnI leak and cardiac function suppression.** (A) After 3 hours of reperfusion, cTnI level was tested with an ELISA kit. (B,C) Cardiac function was assessed by echocardiography in mice 3 days after MI/R injury. MI/R, myocardial ischemia/reperfusion; T siRNA, Tom70 specific siRNA; T scRNA, Scrambled siRNA used as control; cTnI, cardiac troponin-I; LVEF, Left ventricle ejection fraction; LVFS, Left ventricle fractional shortening. Presented values are means ± SEM. N=6 to 8/group. ^*^*P*<0.05 vs. Vehicle of MI/R; ^§^*P*<0.05 vs. T scRNA of MI/R.

**
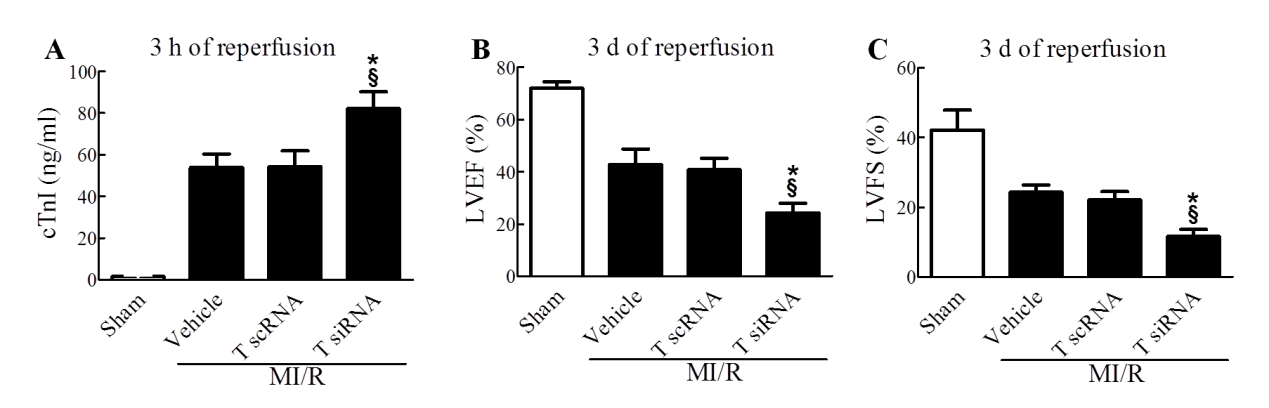
**

**Supplemental Figure7. Tom70 supplementation attenuated I/R-induced cTnI leak and cardiac function suppression via MICU1.** (A) After 3 hours of reperfusion, cTnI level was evaluated with an ELISA kit. (B,C) Cardiac function was assessed by echocardiography in mice 3 days after MI/R injury. MI/R, myocardial ischemia/reperfusion; T sp, Tom70 supplementation; Virus, Lentivirus vector; M siRNA, MICU1 specific siRNA; M scRNA, Scrambled siRNA used as control; cTnI, cardiac troponin-I; LVEF, Left ventricle ejection fraction; LVFS, Left ventricle fractional shortening. Presented values are means ± SEM. N=6 to 8/group. ^*^*P*<0.05 vs. Vehicle of MI/R; ^§^*P*<0.05 vs. Virus of MI/R; ^ψ^*P*<0.05 vs. (T sp + M scRNA) of MI/R.

**
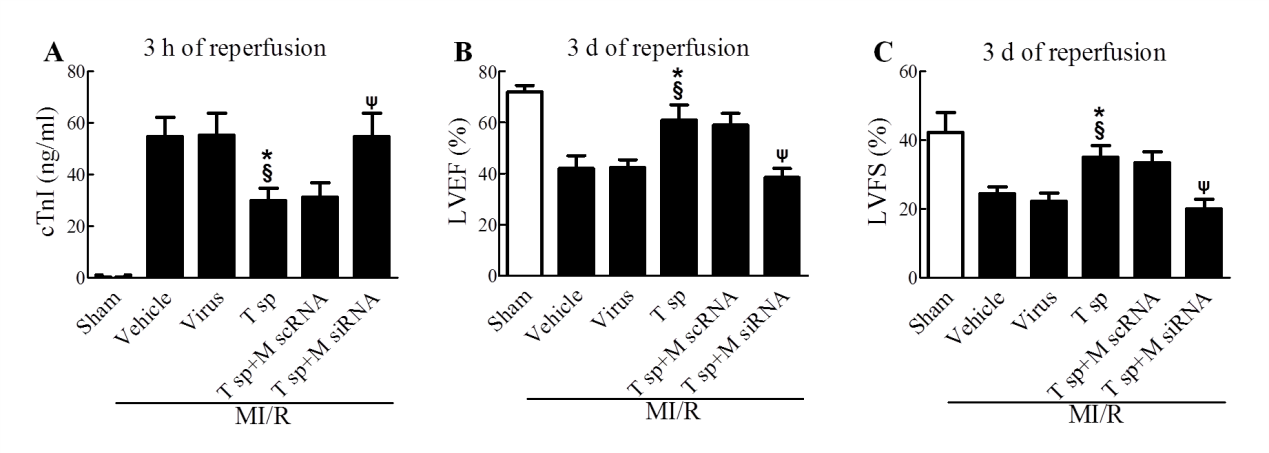
**

**Supplemental Figure8. MICU1 supplementation attenuated MI/R injury depending on Tom70.** (A) Myocardial apoptosis was examined by caspase-3 activity assay. (B) The ATP content in myocardium was determined using a firefly luciferase based ATP assay kit. MI/R, myocardial ischemia/reperfusion; M sp, MICU1 supplementation (the *MIUC1* gene coding sequence was amplified by PCR and subcloned into a lentivirus expression plasmid vector to construct a lentivirus-based overexpression vector carrying the *MIUC1* sequence); Virus, Lentivirus vector; T siRNA, Tom70 specific siRNA; T scRNA, Scrambled siRNA used as control. Presented values are means ± SEM. N=6 to 8/group. ^*^*P*<0.05 vs. Vehicle of MI/R; ^§^*P*<0.05 vs. Virus of MI/R; ^ψ^*P*<0.05 vs. (M sp + T scRNA) of MI/R.

**
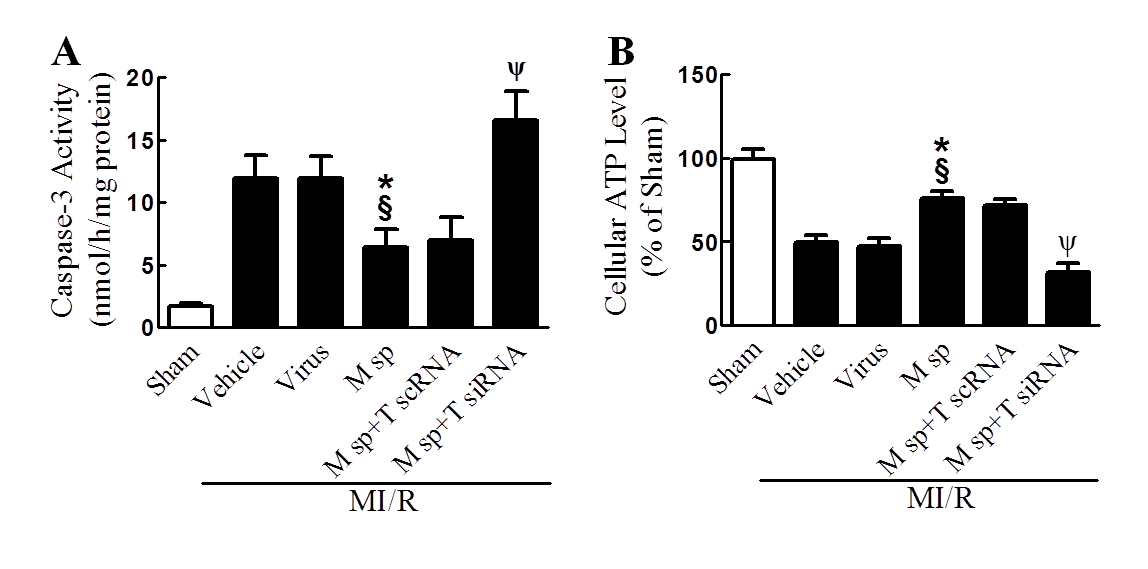
**

**Supplemental Figure9. Schematic diagram of Tom70 and MICU1 in cardioprotection against MI/R injury.** Under normal condition, pre-MICU1s are encoded on the nuclear DNA and synthesized on cytosolic ribosomes after which they are imported into the mitochondria. The translocation of pre-MICU1s must depend on the recognition of Tom complex receptor Tom70. Then MCIU1 exhibits an important role in the mitochondrial Ca^2+^ homeostasis. However, when subjected to MI/R injury, myocardial Tom70 significantly decreases, which suppressed the mitochondrial translocation of MICU1s. At last, mitochondrial Ca^2+^ overload induced by MI/R is deteriorated, contributing to the aggravation of myocardial injury. MI/R, Myocardial Ischemia/Reperfusion; Tom, the translocase of the outer mitochondrial membrane; CM, Cell Membrane; OMM, Outer Mitochondrial Membrane; IMM, Inner Mitochondrial Membrane; MICU1, the mitochondrial Ca^2+^ uptake 1; MCU, the mitochondrial Ca^2+^ uniporter.


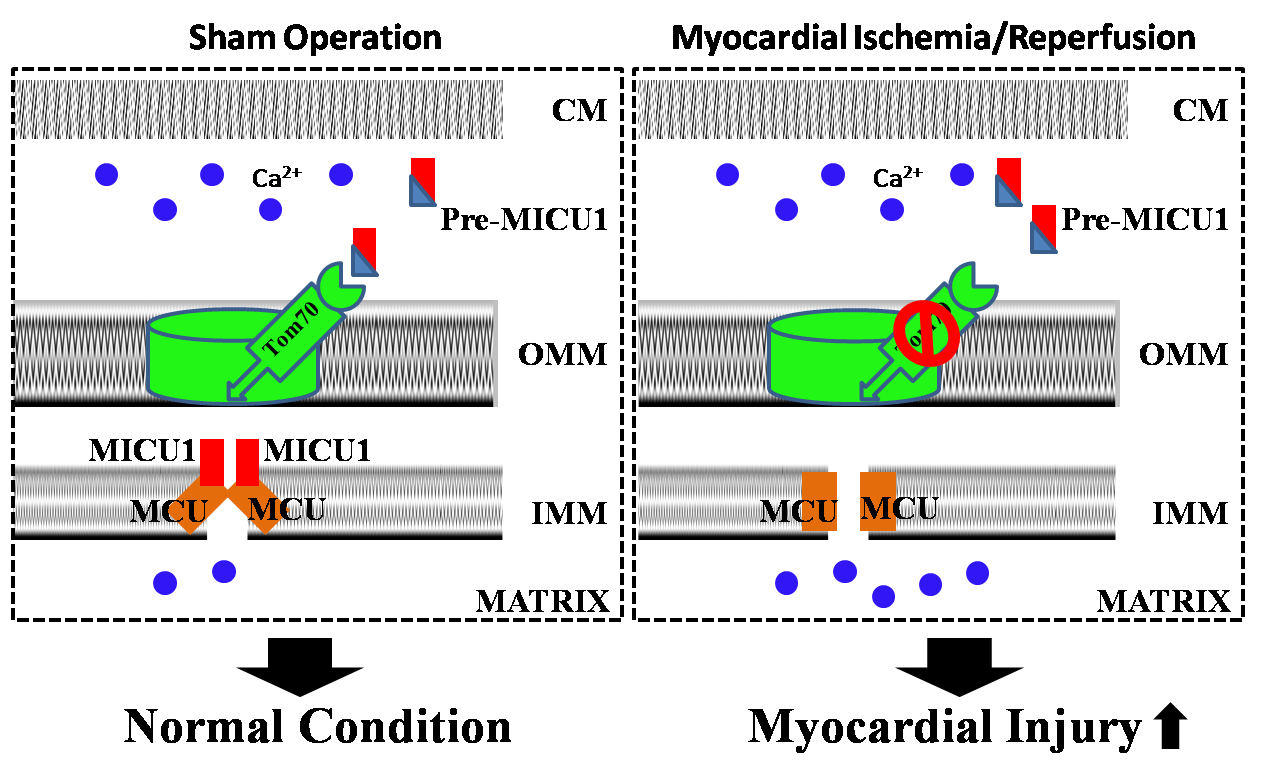

Supplement: Supplementary Information [file cddis2017280x1.docx]
